# The Effect of Partial Substitution of Beef Tallow on Selected Physicochemical Properties, Fatty Acid Profile and PAH Content of Grilled Beef Burgers

**DOI:** 10.3390/foods11131986

**Published:** 2022-07-05

**Authors:** Anna Onopiuk, Klaudia Kołodziejczak, Arkadiusz Szpicer, Monika Marcinkowska-Lesiak, Iwona Wojtasik-Kalinowska, Adrian Stelmasiak, Andrzej Poltorak

**Affiliations:** Department of Technique and Food Development, Institute of Human Nutrition Sciences, Warsaw University of Life Sciences, Nowoursynowska 159c Street, 32, 02-776 Warsaw, Poland; klaudia_kolodziejczak@sggw.edu.pl (K.K.); arkadiusz_szpicer@sggw.edu.pl (A.S.); monika_marcinkowska_lesiak@sggw.edu.pl (M.M.-L.); iwona_wojtasik_kalinowska@sggw.edu.pl (I.W.-K.); adrian_stelmasiak@sggw.edu.pl (A.S.); andrzej_poltorak@sggw.edu.pl (A.P.)

**Keywords:** beef burgers, PAHs (polycyclic aromatic hydrocarbons), fatty acid profile, fat substitutes

## Abstract

The objective of this study was to analyze the impact of partial replacement of beef tallow with sunflower, canola, linseed, olive oil and milk fat on physical properties, oxidation stability, fatty acid profile and PAHs (polycyclic aromatic hydrocarbons) content of beef burgers. Studies have shown a strong relationship between the fatty acid profile and the PAH content (especially of the heavy PAHs). The partial replacement of beef tallow with oils and milk fat (MF) contributed to a change in the fatty acid profile and a reduction in the hardness of the burgers. The highest PAH content was found in samples with canola oil (CO), which had the highest levels of monounsaturated fatty acids (MUFA), and in the control group (CON) without fat substitution, which had the highest levels of saturated fatty acids (SFA) and trans conformations. Substitution of animal fat with vegetable oils contributed to a change in the color of the burgers’ surface, as there was a statistically significant increase in the L* color component and a decrease in the a* component. The burgers with canola oil (CO) and linseed oil (LO) were the most susceptible to oxidation, whereas the burgers with reduced fat content (CON_LOW FAT) were the most stable in terms of oxidation, where the malondialdehyde (MDA) content was 32.8% lower compared with the control group (CON). The studies confirm that partial replacement of beef tallow with vegetable oils and milk fat and reduction in fat content in burgers to be grilled can be an effective way to change their fatty acid profile and reduce the cyclization reaction of organic compounds leading to the formation of PAH. Correlation coefficient analysis showed that there is a relationship between fatty acid profile and the presence of selected PAHs in grilled beef burgers. The results of this study indicate that replacing beef tallow with vegetable oils is a promising approach in designing meat products with controlled PAH content.

## 1. Introduction

Quality and safety are key issues in food production and processing. Meat is processed to obtain suitable functional properties. Heat treatment confers desirable sensory characteristics to meat products and extends shelf life [1]. However, this treatment also results in the formation of harmful molecules such as polycyclic aromatic hydrocarbons (PAHs).

Grilled food presents a unique taste and aroma [2], but the formation of PAH depends on many factors. Among the most important are the type of raw material, the fat content of the product, the food additives used, the type of fuel used, the heat treatment method chosen, the time and temperature of the process, the positioning of the product relative to the heat source, and the condition and construction of the equipment [3,4].

The fat content in food is directly determinant of PAH contamination in grilled products [5]. Animal fat used in the food industry plays an important role (as a carrier of taste) in creating attractive sensory characteristics. At the same time, animal fats are perceived negatively due to their high content of cholesterol and saturated fatty acids. A correlation has been shown between high consumption of animal fats and obesity, and certain types of neoplasms and cardiovascular diseases [6]. Therefore, modifying the composition of meat products to reduce or partially replace fat appears to be a suitable strategy to reduce the risk of PAH formation and improve the nutritional value of animal products.

One of the most popular meat products used for grilling is burgers as confirmed by the study conducted by Szpicer et al. [7] who analyzed the possibility of producing low-fat beef burgers by replacing beef fat with canola oil and oat beta-glucan concentrate. The study showed favorable effects of the modification on water holding capacity and cholesterol levels in burgers, whereas maintaining high consumer acceptance. The modifications made did not affect the texture parameters in any significant way. Replacing part of the beef tallow with rapeseed oil caused a reduction in the values of the a* and b* parameters, whereas not significantly affecting the value of the L* parameter. A study by Afshari et al. [8], in which beef fat removed from meat was replaced with a mixture of vegetable fats, contributed to enhancing the nutritional and processing properties of beef burgers. The researchers used an emulsion consisting of canola oil, olive oil, soy protein isolate, and water. Breadcrumbs were substituted by a mixture of beta-glucan and inulin. The changes in fat content resulted in an improved fatty acid profile and increased hardness, but this effect was compensated by the addition of beta-glucan and inulin, so that consumer acceptance of the product was not adversely affected.

The aim of this study was to investigate the effect of partial replacement of beef tallow with sunflower oil (SO) canola oil (CO), linseed oil (LO), olive oil (OO) and milk fat (MF) on selected physicochemical parameters (proximate composition, L*, a*, b* color, texture, oxidative stability), fatty acid profile, and PAH content in grilled beef burgers.

## 2. Materials and Methods

### 2.1. Sample Preparation and Grilling Procedure

Beef neck and tallow were purchased from the local factory (Meat Plant Wierzejki Ltd., Trzebieszów, Poland) and ground using a meat mincer with a Ø 8 mm plate (PI-22-TU-T meat mincer, Edesa, Spain). Burgers were prepared for seven different treatments (determined during pilot experiments). Table 1 presents the composition of the control group burgers (CON), burgers with fat reduced by 48.2% (CON_LOW FAT), burgers with sunflower oil (SO), canola oil (CO), linseed (LO), olive oil (OO) and milk fat (MF).

Burgers with ingredients according to a provided recipe have been grilled using the Weber Master-Touch Premium GBS E-577 grille. During the heat treatment, temperature was monitored by means of a digital meter (Testo 926, Lenzkirch, Germany). The temperature of charcoal was between 280 and 300 °C. During grilling, all samples were flipped every 3 min. The charcoal was exchanged for new one between grilling of the different research groups, which was important especially in terms of PAH profile analysis. Once 75 °C was reached at the geometric center. The samples were removed from the grill and subjected to relaxation for 15 min at room temperature. After temperature equilibration, the samples were cooled to 4 °C, then vacuum-packed in polyethylene bags (Cryovac^®^ VS26, Sealed Air Corporation, Elmwood Park, NJ, USA) and stored at 4 °C. Twelve burgers were prepared in each group, of which 6 pieces were tested on day 1 and 6 on day 6. The samples protected in this way were the test material for the measurement of proximate composition, L*a*b* color, textural properties, oxidative stability as per lipid oxidation analysis (TBARS), fatty acid profile and 12 PAHs. The test material was also secured in the form of raw burgers, which were vacuum packed and tested on day 1 and 6. After 6 days, the burgers were unpacked from the vacuum bags, heat treated (by grilling) and then L*a*b* color, textural properties, TBARS, fatty acid profile and PAHs were measured. The grilling process parameters on day 1 and day 6 were the same.

### 2.2. Chemical Composition Analysis

The proximate composition of the grilled burgers on day 1 was determined using near-infrared spectroscopy with a Büchi N-500 NIRFlex spectrometer (Büchi Labortechnik AG, Flawil, Switzerland) with the module NIRFlex Solids. Measurements were carried out in the spectral range 12,500–4000 cm^−1^, in reflectance. The homogenized sample was scanned in the measuring module of the instrument. The measurement was repeated three times for each sample and the water, protein, fat, ash and connective tissue contents in percentage were presented as arithmetic means [9].

### 2.3. Color Measurement

CIE (Comission Internationale de l’Eclairage) L*a*b* color analysis was performed using the reflection method using the Minolta CR-400 colorimeter (Konica Minolta Inc., Tokyo, Japan). The instrument was calibrated using a white standard (L* = 98.45, a* = −0.10, b* = −0.13). The following parameters were measured: L* (lightness), a* (from −a* “greenness” to +a* “redness”) and b* (from −b* “blueness” to +b* “yellowness”). Measurements were made on the burgers’ surfaces before and after grilling on days 1 and 6 of storage. The results are presented as an arithmetic mean of 10 measurements.

### 2.4. Texture Measurement

Texture profile of the grilled burgers was analyzed using an Instron 5965 universal strength testing machine (Instron, Norwood, MA, USA). A double compression test was used in which the samples were compressed until reaching 50% of their initial height. The relaxation time was 3 s, and the head speed was 200 mm/min. The analysis was performed on samples of uniform shape and dimensions (diameter = 25.4 mm, height 25 mm). The measurement was performed 6 times for each test group of day 1 and day 6. Parameters such as springiness (–), chewiness (N) and hardness (N) were determined from the force-time curve using the procedure described in the study by Półtorak et al. [10].

### 2.5. Lipid Oxidation Analysis (TBARS)

Burger samples weighing approximately 5 g were soaked in 50 mL of TCA (trichloroacetic acid) solution (20% trichloroacetic acid solution acidified with 1.6% concentrated phosphoric acid, cooled to 4 °C) and 2.5 mL of antioxidant (antioxidant: aqueous-ethanol 1:1 solution containing 0.5% propyl gallate and 0.5% EDTA) was added [11]. They were homogenized for 2 min at 12 rpm × 1000 (Ultra Turrax homogenizer, IKA T18 basic, Königswinter, Germany). The whole product was filtered through Whatman grade 1 filter paper and made up to 100 mL with water-ethanol mixture (1:1). A 5 mL sample was taken and 5 mL of 0.02 M TBA (thiobarbituric acid) solution was added. The samples were incubated for 40 min in a boiling water bath (WNB 7 Memmert, Büchenbach, Germany). Simultaneously, a sample blank was prepared in which the meat sample was replaced with a TCA: water mixture (1:1). Spectrophotometric measurement was carried out at a wavelength of 532 nm. TBARS (thiobarbituric acid reactive substances) concentrations were calculated using 1,1,3,3-tetramethoxypropane (TEP, 0–11 µM) as standard. The TBARS value was expressed as milligram of malondialdehyde (MDA) produced per kilogram of sample (mg MDA/kg sample). Grilled burgers were tested on days 1 and 6 of storage.

### 2.6. Fatty Acids Profile Analysis

The fatty acid profile of raw beef burgers from days 1 and 6 was determined by gas chromatography. Fat from the test samples was extracted with a chloroform-methanol mixture as per the procedure by Folch et al. [12]. The resulting fatty acid methyl esters (FAME) were analyzed using a Shimadzu GC-2010 gas chromatograph with a flame ionization detector (FID) equipped with a RT^®^ 2560 silica column (100 m × 0.25 mm ID and 0.2 µm film thickness) (RESTEK, Bellefonte, PA, USA). Samples of 1 mL were analyzed in three repetitions. Compounds were identified by comparing the retention times obtained with those of fatty acid standards (Supelco^TM^ 37 Component FAME mix, Sigma, St. Louis, MO, USA). The results were averaged and expressed in grams per 100 g fat.

### 2.7. Standards and Calibration Solution (PAHs)

The standards of fluorene (F), anthracene (ANT), fluoranthene (FL), benzo[b]fluorine (BbF), benz[a]anthracene (BaA), chrysene (CHR), benzo[b]fluoranthene (BbFL), benzo[k]fluoranthene (BkF), benza[a]pyrene (BaP), diben[a,h]anthracene (DBahA), benzo[g,h,i]perylene (BghiP), indeno[1,2,3-cd]pyrene (IP) obtained from Perlan Agilent Technologies (Agilent Technologies, Santa Clara, CA, USA). Stock solutions of each PAH were prepared by dissolving 0.01 g of standards in high purity acetonitrile (Sigma–Aldrich, Darmstadt, Germany). Calibration solutions were then prepared by serially diluting the stock solutions into a five-point concentration range of 0.05–20.0 µg/kg.

### 2.8. PAH Extraction and Quantification

The PAHs were extracted by the method described by Bogdanović et al. [13]. The first step was a reaction, in which lipids were saponified by incubating 10 g of homogenized samples in potassium hydroxide solution in 1 M ethanol (25 mL) in a water bath (WNB 7 Memmert, Büchenbach, Germany) at 80 °C for 2 h. The samples were then moved to separatory funnels and extracted three times with 15 mL cyclohexane, which was then evaporated. The next step was a two-step sample purification by SPE as described in detail by Kafouris et al. [14]. For the purification, a C-18 column was used first, which was activated with methanol (24 mL) and acetonitrile (24 mL). Further description of the method is given in the study by Onopiuk et al. [15].

The determination of 12 polycyclic aromatic hydrocarbons was carried out by high performance liquid chromatography with fluorescence detector (Analytical HPLC, 1260 Infinity II LC System, Agilent Technologies, Santa Clara, CA, USA). The separation was carried out on an appropriately sized Agilent ZORBAX Eclipse PAH column, with dimensions 4.6 mm × 150 mm, 3.5 µm, and flow rate 1.3 mL/min. The moving phase consisted of water (A) and acetonitrile (B), column temperature was 25 °C, injection volume was 5 µL.

### 2.9. Statistical Analysis

Data were statistically analyzed using Statistica 13.1 (StatSoft Inc., Tulsa, OK, USA). One-way analysis of variance was performed using Tukey’s test at a significance level of *p* < 0.05. Results are presented as mean values with their standard deviation (SD).

## 3. Results and Discussion

### 3.1. Chemical Composition Analysis

Proximate composition of grilled burgers on the 1st and 6th day of storage is shown in Table 2.

The burgers with olive oil (OO) showed the highest moisture value on day 1 (54.7%) whereas the canola oil group (CO) had the lowest water content value (49.1%) (*p* < 0.001). On day 6, the control group with reduced fat content had the highest water content (59.25 ± 0.04%) and burgers from the groups with sunflower oil (51.26 ± 0.15%) and linseed oil (51.83 ± 0.30%) had the lowest. Most of the groups experienced statistically significant (*p* ≤ 0.05) reduction in moisture content during storage. The highest fat content on day1 was present in samples with sunflower oil (15.27 ± 0.26%), the lowest was recorded in samples belonging to CON_LOW FAT (12.16 ± 0.09%), OO (12.23 ± 0.16%) and MF (12.01 ± 0.11%) groups. On day 6, statistically significantly (*p* ≤ 0.05) the lowest fat content was observed in the reduced fat control group (10.28 ± 0.16%). At the same time, the highest protein content, at 30.37 ± 0.15%, was observed in this group. Burgers with linseed oil contained the lowest proportion of protein in the proximate composition, which was 26.21 ± 0.26%. When analyzing the salt content of the burgers, the highest salt content was found in the control group (1.42 ± 0.04%) and the lowest salt content was found in the burgers with sunflower oil (0.45 ± 0.02%) and canola oil (0.61 ± 0.02%). A statistically significant (*p* ≤ 0.05) reduction in the percentage salt content of the samples was observed in most groups during storage. The samples with milk fat (MF) contained the lowest level of connective tissue on day 1 and day 6 (day 1 3.60 ± 0.06%; day 6 1.82 ± 0.09%) and the samples from CON group contained the highest level. As for the connective tissue content, its percentage decreased statistically significantly (*p* ≤ 0.05) during storage in each group.

The proximate composition is a very important parameter because it can directly affect the physical properties of burgers (springiness, chewiness, hardness), fatty acid profile and PAH formation during grilling [16]. According to studies conducted by Trujillo-Mayol et al. [17], burger composition also determines the amount of heterocyclic aromatic amines and acrylamide formed during burger frying. A key parameter affecting the amount and profile of PAHs is the total fat content, as confirmed by Wongmaneepratip and Vangnai [18] in their study. The analysis of water content led to the conclusion that in low-fat burgers and those containing pre-emulsified vegetable oils, moisture retention is attributed to the stabilizing effect of oil in an established emulsion system, as confirmed and reported in the study by Afshari et al. [8]. The significantly lower protein content in burgers with vegetable oil (CO, LO, OO) was due to the partial substitution of beef tallow. Scientific reports indicate a significant protein content in animal fat and the results obtained are confirmed in the literature [19,20]. The diversity between the groups in terms of fat content was related to differences in the fatty acid profile, technological properties and production method of the beef tallow substitutes used. Ingredients available in local stores were used in the experiment. Canola oil and sunflower oil as a partial substitute of beef tallow resulted in increased fat content in the samples. These oils are commonly used in the preparation of heat-treated foods, which is associated with a properly adapted production process to ensure the desired technological properties (high thermal hydrolysis point temperature). Olive oil and linseed oil, used in meals consumed without heat-treatment, are characterized by different properties, which led to a reduced fat content in the samples compared with the control, CON_LOW FAT, SO and CO groups, which could be explained by increased fat leakage during thermal processing.

### 3.2. Color Measurement

The CIE L*a*b* color components for raw and grilled burgers on the 1st and 6th days of storage are shown in Table 3. It was found that 6 days of storage had no statistically significant (*p* > 0.05) effect on the color change in burgers, either raw or grilled. Among raw burgers, the value of parameter L* (darkest) was lowest for samples from the control group (day 1 46.65 ± 2.18) and the control group with reduced fat content (day 1 47.66 ± 2.33). These values were statistically significantly (*p* ≤ 0.05) lower than in samples where beef tallow was partially replaced with other fats. Among all groups of raw burgers, the highest value of parameter L* (brightest) was recorded for samples with sunflower oil (day 1 52.73 ± 2.92). The parameter a* of raw burgers did not differ statistically significantly, neither between 1 and 6 days of storage, nor between the test groups with different recipes. The lowest values of the parameter b* were found in samples of the control group (day 1 10.46 ± 1.85) and the reduced-fat control group (day 1 13.15 ± 2.12). The parameter b* differed statistically significantly (*p* ≤ 0.05) between the control groups, whereas no statistically significant differences were observed between the remaining test groups, of which the highest value of the parameter b* was found for the sample with linseed oil (day 1 16.41 ± 1.64). In Foggiaro et al.’s [21] study, pork back fat in burgers was replaced by walnut and algal oil mixture hydrogel or pistachio and algal oil mixture hydrogel. The change in recipe resulted, as in the present study, in a significant increase in the values of the L* and b* parameters in the raw burgers [21]. The increased proportion of the b* component is probably due to natural differences between vegetable fats and milk fat, characterized by their yellow color, and beef tallow, which has a color more similar to white. A higher proportion of the yellow component may also be related to a higher degree of lipid oxidation in samples with vegetable oils [22,23].

The highest value of parameter L* of heat-treated burgers occurred in the samples from the control group (day 1 = 35.32 ± 0.87) and samples with olive oil (day 1 = 34.17 ± 1.67). The lowest value of the parameter L* was recorded for the samples with sunflower oil (day 1 = 28.09 ± 2.77) and milk fat (day 1 = 27.64 ± 2.08). The parameter a* reached the lowest values in samples from the control group with sunflower oil and milk fat (7.26–7.30 on day 1), and these values did not differ statistically significantly (*p* > 0.05). The SO (day 1 8.51 ± 2.04) and MF (day 1 8.72 ± 1.29) groups had the lowest value of component b*, whereas the CON group had the highest value (11.94 ± 0.62 on day 1 and 11.69 ± 0.56 on day 6). The results showed that the reduction in beef tallow content or its partial replacement with other fat affects the color of raw and grilled burgers. The lower value of L* and b* parameters in grilled burgers in which animal fat was replaced by another ingredient is confirmed by a study by Botella-Martinez et al. [24] where 50% or 100% of the animal fat in the samples was replaced by a gelled emulsion elaborated with cocoa bean shell flour and walnut oil. Similar conclusions were reached in the study by Wongmaneepratip and Vangnai [18], where the color of marinated and then grilled chicken breasts was measured. The addition of palm oil and sunflower oil to the marinades resulted in a significant change in color components L* and a* compared with the control group. The results obtained in this study are comparable to the burger color presented by Heck et al. [25] The authors also confirmed that heat treatment contributes to the reduction in L* and a* color components. A study conducted by Szpicer et al. [7] confirmed that the addition of canola oil to beef burgers causes a significant decrease in the parameter b* (*p* < 0.05). The increase in parameter L* in samples with vegetable oils can be explained by the fact that meat emulsions with such oils had a much smaller oil globules, which reflect more light (have larger surface area) than larger animal fat globules. Similar conclusions were reached in the studies by Youssef and Barbut [26] and Selani et al. [27]. A similar relationship was obtained in their study by Rodríguez-Carpena et al. [28], where they added avocado, sunflower and olive oils to pork burgers. The observed color differences between raw and grilled samples are related to the several changes that occur during thermal processing. These include the Maillard reaction, denaturation of proteins, and loss of fat and water. Grilling led to an increase in the variability of the proportions of color components between the test groups due to the different properties of the beef tallow substitutes used to prepare the burgers [29].

### 3.3. Texture Measurement

The springiness, chewiness, and hardness measured in the grilled burgers are presented in Table 4.

From the analysis of the results of textural properties, it can be concluded that the modifications introduced in the burger recipe resulted in the greatest changes in parameters such as chewiness and hardness (both during storage and between burger groups). On the other hand, the springiness parameter did not change much and was in the range of 0.66–0.68. The greatest difference in springiness was observed only for the control group with reduced fat content, where this value decreased in a statistically significant manner (*p* ≤ 0.05) from 0.66 on day 1 to 0.59 on day 6. Similarly, for chewiness: the samples from the three groups had the lowest hardness on day 1 in a statistically significant (*p* ≤ 0.05) manner: LO (34.99 ± 3.23 N), OO (39.87 ± 4.61 N) and MF (45.62 ± 1.78 N). These values were about twice as low as the highest value recorded for the reduced-fat control group (88.64 ± 9.82 N). On day 6, the lowest hardness value was observed in the canola oil group (45.71 ± 5.95 N). The highest in the control groups (CON_LOW FAT 76.72 ± 12.79 N; CON 74.88 ± 7.99 N). Recipe modifications had a positive effect on reducing the hardness of burgers, as confirmed by the study by Szpicer et al. [7]. The replacement of beef tallow with canola oil resulted in a 15.39% decrease in hardness compared with the control group containing 21.42 ± 0.26% fat. A decrease in hardness due to replacement of animal fat with vegetable oils was also observed in the study by Lee et al. [30]. The lack of effect of fat substitution on the elasticity parameter was described by de Oliveira Fagundes et al. [31], where the authors replaced animal fat with canola oil gels. The increased hardness of burgers with vegetable oils, i.e.,: canola oil and sunflower oil may be due to the lower fat globule of vegetable fat when compared with animal fat and the resulting higher protein–protein and protein-lipid interaction [22]. Since fat imparts flavor, tenderness, and juiciness to food products, reducing it leads to harder products, as confirmed by Selani et al. [32]. Additionally, Heck et al. [25] confirmed that the hardness of burgers increases with increasing levels of animal fat substitutes, which were hydrogelated emulsions of chia and linseed oils.

### 3.4. Lipid Oxidation Analysis (TBARS)

**Figure 1:**
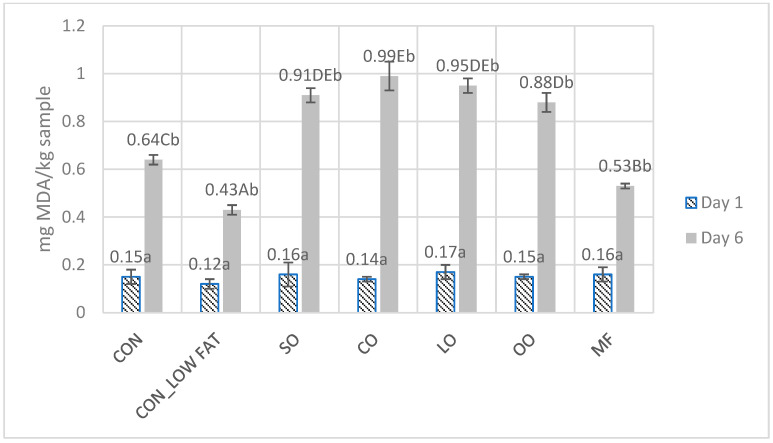
The lipid oxidation degree in burgers on the 1st and 6th day of storage measured by TBARS method. CON—control group beef burgers, CON_LOW FAT—burgers with reduced fat, burgers with fat substitutions, i.e., sunflower oil (SO), canola oil (CO), linseed (LO), olive oil (OO) and milk fat (MF). (A–E)—means with different letters showing significant effect of treatment group in the same day of storage; a, b—means with different letters showing significant effect of storage time in each treatment group; *p* ≤ 0.05.

The degree of lipid oxidation in the prepared raw burgers was examined using thiobarbituric acid and malondialdehyde (Figure 1). On the first day of testing, samples from all groups showed similar levels of lipid oxidation, ranging from 0.12–0.17 mg MDA/kg of sample. On the 6th day of storage, there was a statistically significant (*p* ≤ 0.05) increase in the degree of lipid oxidation in each of the analyzed groups. The highest degree of lipid oxidation was observed in canola oil group (0.99 ± 0.06 mg MDA/kg), SO group (0.91 ± 0.03 mg MDA/kg) and LO group (0.95 ± 0.03 mg MDA/kg). The highest stability to oxidation was present in burgers with reduced fat content (0.43 ± 0.02 mg MDA/kg), where oxidation was about 32.8% slower compared with the control group. Modification in the fat used significantly affected the fat oxidation level after 6 days of storage. The use of vegetable oils (SO, CO, LO, OO) increased the degree of lipid oxidation compared with samples containing animal fats (CON, CON_LOW FAT, MF).

The reduction in lipid oxidation due to reduced fat content in burgers is supported by the study of Patinho et al. [33]. The authors investigated the possibility of replacing some pork fat in beef burgers with *Agaricus bisporus* (AB) mushrooms (5%, 10%, 15% AB and 15%, 10% and 5% pork fat, respectively). In this study, TBARS values increased during storage, but the burgers with fat content reduced by replacing it with common mushrooms had significantly lower TBARS values than the control samples. The TBARS values in the group in which half the fat was replaced with common mushrooms were similar to those obtained in the CON_LOW FAT group in this study and were approximately 0.4 mg MDA/kg. The experiment showed that the addition of common mushrooms resulted in higher oxidative stability and moisture retention, lower fat content, and reduced cooking losses. The obtained product was characterized by good technological quality and desirable sensory attributes.

A study conducted by Szpicer et al. [7], where the effect of replacing 40% of beef tallow with canola oil on the TBARS value in beef burgers was analyzed, confirmed, similar to this study, that replacing animal fat with vegetable oil can cause an increase in oxidative processes both on the first day of testing and during storage. The increase in TBARS values due to replacement of animal fat with vegetable fat was also confirmed in the study by Heck et al. [25], where beef burgers with 40% replacement of pork fat by a hydrogel emulsion of chia oil and linseed oil had higher lipid oxidation from 0.27 to 0.53 mg MDA/kg.

A study by Trujillo-Mayol et al. [17] showed that an effective method to reduce the degree of lipid oxidation in beef burgers stored for 10 days can be the addition of avocado peel extract to the burger recipe. This is because plant extracts, which are rich in phenolic compounds, have the ability to limit the oxidative reactions in meat products. According to Lu et al. [34], the susceptibility of acids to oxidation increases in proportion to the number of unsaturated bonds in each fatty chain. Oils with high linolenic and linoleic acid content, i.e., linseed oil and sunflower oil, have the highest susceptibility to oxidation. The oxidative stability of olive oil, similarly to canola oil, is much higher due to its high oleic acid content. In conclusion, the use of beef tallow substitutes characterized by high unsaturated fatty acid content leads to increased lipid oxidation and thus to higher MDA content in the samples during storage. This is confirmed in the scientific literature in studies by Botella-Martínez et al. [29], Heck et al. [22], Lucas-Gonzalez [35] and Moghtadaei [36], among others.

### 3.5. Fatty Acids Profile Analysis

The fatty acid profile of the samples grilled on the 1st and 6th day of storage was analyzed. The mean proportions of each fatty acid, the content of total saturated (SFA), monounsaturated (MUFA) and polyunsaturated fatty acids (PUFA), and the PUFA/SFA and n6/n3 ratio are shown in Table 5 and Table 6.

The saturated fatty acid content was highest on both day 1 and day 6 in the group, in which beef tallow was replaced by milk fat (day 1 48.76%; day 6 49.77%). The lowest SFA value on day 1 was observed in samples with canola oil (26.53%), similar to day 6 (27.48%). Similar SFA values were also obtained for groups with sunflower oil, linseed oil and olive oil. Analyzing the individual saturated fatty acids, the statistically significantly (*p* ≤ 0.05) highest content of C12:0, C14:0, C15:0 and C:16:0 fatty acids was recorded in the group with milk fat (MF on day 1 and day 6). The highest C20:0 eicosanoic acid content was observed in the MF group (day 1 = day 6 = 0.67) and the lowest in the LO group (day 1 = 0.32; day 6 = 0.34). In most cases, no statistically significant differences were observed between day 1 and day 6 except for C14:0 fatty acid in the CON, CON_LOW FAT, SO and OO groups and C16:0 fatty acid in the OO group, where values on day 1 were significantly (*p* ≤ 0.05) higher than on day 6.

The lowest statistically significant (*p* ≤ 0.05) content of total monounsaturated fatty acids on day 1 and day 6 was observed in the linseed oil burger group (day 1 33.66%; day 6 34.02%). These burgers had the lowest content of all monounsaturated fatty acids (MUFA) on day 1 and day 6. The only exception to this was C18:1 *trans* acid, which was at its lowest level of 0.02% in the burgers from the LO group. The MUFA found to have the highest amount in the burgers was C18:1 *cis* acid, and its highest value was present in the burgers with olive oil (58.83%). No C24:1 fatty acid was detected in any study group except the CO group (day 1 = day 6 = 0.04%).

The group with linseed oil added had the highest (day 1 38.52%, day 6 37.09%), statistically significant (*p* ≤ 0.5), polyunsaturated fatty acid (PUFA) content. The lowest PUFA contents on day 1 were observed in the CON_LOW FAT and MF groups. Of the polyunsaturated fatty acids, linoleic acid (C18:2 9.12 *cis*) was of the highest amount, with the highest content on days 1 and 6 present in the linseed oil group (day 1 34.37%, day 6 33.22%). By analyzing the PUFA to SFA ratio, it was observed that PUFA dominated in the LO (1.37) samples on day 1 and SO (1.03) and LO (1.28) samples on day 6. The significantly statistically highest ratio of n6 to n3 fatty acids was observed in the sunflower oil group (day 1 57.8 and day 6 92.76). The other study groups had similar n6/n3 ratio values, ranging from 3.79 to 10.75 on day 1 and from 3.92 to 10.99 on day 6. Analyzing the differences between the KT profile on day 1 and day 6, it was observed that SFAs had the highest stability during storage, whereas PUFAs had the lowest stability (they oxidize the fastest).

The partial replacement of beef fat in the burgers contributed to a decrease in saturated fatty acids and an increase in MUFAs and PUFAs, which is consistent with previous studies by Szpicer et al. [37]. The study then showed that replacing beef fat with canola oil and oat beta-glucan concentrate improved the fatty acid profile. Another method of changing the fatty acid profile of burgers was used by Carvalho et al. [38], where the fat content of the base composition was reduced by adding wheat fiber at 1.25, 2.50, 3.75, and 5.00 g per 80 g serving of burger (the fiber was hydrated before being added to the rest of the ingredients). The pork fat was reduced so that the 4:1 meat-to-fat ratio was maintained. Only the addition of the greatest amount of fiber negatively affected consumer acceptance of the burgers. The other test groups were evaluated positively and showed no or statistically insignificant changes in process-specific and sensory characteristics compared with the control group. As in the study conducted by Afshari et al. [8], the applied lipid modification improved the fatty acid profile by reducing the percentage of SFA and lowering the n6/n3 acid ratio in CON_LOW FAT, CO and MF groups. The addition of fiber (a mixture of 3.1% inulin and 2.2% β-glucan), canola oil and olive oil as substitutes for beef fat in the study of Afshari et al. [8] reduced SFA levels from 48% (control group) to about 19–24% and reduced the n6/n3 ratio from 8.6 to about 3. Our study confirmed that replacing animal fat with canola oil, sunflower oil, and linseed oil increases PUFA concentrations due to the presence of linoleic acid (C18:2 n-6) and linolenic acid (C18:3n-3) as the major fatty acids of sunflower oil, canola oil, and linseed oil, respectively. According to the study by Selani et al. [32], the addition of canola oil to the formulation may contribute to the reduction in fatty acids such as myristic, palmitic and stearic acids compared with other vegetable fats.

### 3.6. The Effect of Fat Replacement on the Formation of PAHs

According to Wongmaneepratip and Vangnai [18], the specific mechanism of PAH formation is not well known, but some researchers have suggested that these compounds may be formed in free-radical reactions, intramolecular addition reactions or polymerization of small organic molecules.

Samples of burgers grilled on the 1st and 6th day were analyzed to find the profile of polycyclic aromatic hydrocarbons. The content of individual PAHs, the sum of 12 PAHs, the sum of the light compounds, and the sum of heavy compounds are given as arithmetic means in mg/kg for a sample (Table 7). As anticipated, it was statistically significant (*p* ≤ 0.05) that the lowest level of Σ12 PAHs was present for low-fat samples (CON_LOW FAT), where on day 1, the total of 12 PAHs was at 48.46 mg/kg and in day 6 47.68 mg/kg. The highest statistically significant level (*p* ≤ 0.05) of PAH was observed in samples with canola oil (day 1 105.5 mg/kg: day 6 102.69 mg/kg). The analysis of the content of the individual light PAHs showed that the group with canola oil showed the highest levels of fluorene (day 1 0.94 ± 0.12 mg/kg), anthracene (day 1 1.49 ± 0.25 mg/kg), chrysene (day 1 22.11 ± 0.56 mg/kg) and benzo[b]fluorine (18.86 ± 0.43 mg/kg) on day one.

The highest content of heavy PAHs was recorded in canola oil samples (day 1 60.75 mg/kg, day 6 59.56 mg/kg). This group showed statistically significantly (*p* ≤ 0.05) highest contents of 4 heavy PAHs (BbFL 2.47 ± 0.23 mg/kg, BkF 4.86 ± 0.31 mg/kg, DBahA 46.97 ± 1.06 mg/kg, BghiP 3.15 ± 0.30 mg/kg) on both test days. The lowest benzo[a]pyrene content in a statistically significant manner (*p* ≤ 0.05) was found in burgers from the reduced fat group (1.23 ± 0.21 mg/kg). Indeno[1,2,3-cd]pyrene was not detected in any of the study groups. Analysis of changes in PAH content between day 1 and day 6 showed that statistically significant changes (*p* ≤ 0.05) were least frequent in the reduced-fat group and most frequent in the OO and CO groups.

The PAH results presented in this study are comparable to the results obtained in Onopiuk et al. [39], where levels of individual PAHs as well as their total are presented for charcoal-grilled pork. Slightly higher BaA, BaP, BaF values for grilled pork tenderloin were obtained in by Cordeiro et al. [40], where they studied the effect of using elderberry vinegar, white and red wine vinegar, cider vinegar, and fruit vinegar with raspberry juice, as marinades, on PAH formation during high-temperature heat treatment. There is great difficulty in discussing and directly comparing PAH results between studies. This is mainly due to the large number of factors affecting the formation of PAHs such as: type of meat, fat content, presence of other ingredients, heat treatment conditions and many others [2]. The mechanism of PAH formation, which is not fully understood, is also an important difficulty, whereas studies confirm a strong relationship between fat content and type of fat and PAH levels in food products. In a study by Lu et al. [34], 40% of pork fat was replaced with sunflower oil, olive oil or grape seed oil. The researchers demonstrated that oil type has an effect on PAH content, and the direction of these changes exhibits differences that depend on the heat treatment temperature used. The use of olive oil reduced the PAH content by more than 50% compared with the control group for a temperature of 220 °C. In the present study, a reduction in PAH levels due to the use of olive oil of about 30% was achieved. In the study by Hu et al. [41], beef was injected with the following fats: colza oil, soybean oil, canola oil, sunflower oil, butter, and pork fat. The research findings showed that the PAH content increased significantly due to the use of canola oil, which is consistent with the results obtained in this study. Hu et al. [41] also showed that the total PAH content of the samples with pork fat was significantly higher than in the samples with butter, similar to the MF burgers in the present study. The use of fat replacements for heat-treated meat products can be an effective way to reduce the levels of cyclic organic benzene derivatives. Natural vegetable oils such as olive oil and linseed oil contain fatty acids that play a role in the fragmentation of hydrocarbons as well as the cyclization of aromatic compounds. The lowest PAH content in the CON_LOW FAT group may be due to the lowest fat and highest water content. Research by Kafouris et al. [14] confirm a strong positive correlation between fat content and PAH formation in meat products. The highest PAH levels in the canola oil burger group can be linked to the fastest lipid oxidation process in this group. According to Lu et al. [42], lipid oxidation and protein oxidation are associated with the development of HCA and PAHs through the interaction of radicals generated by lipid oxidation, lipid pyrolysis and Maillard reaction during the heat treatment.

The correlation coefficients between the profiles of selected fatty acids of raw burgers and PAHs of grilled burgers are presented in Table 8 (selected highest correlation coefficients are shown in the table). Based on the results, high correlation was found between fluorene, benz(a)anthracene, benzo[b]fluoranthene and benzo[k]fluoranthene content and fatty acids such as γ-linolenic acid GLA (C18:3, 6, 9, 12), eicosenoic acid (C20:1), erucic acid (C22:1) and nervonic acid (C24:1). Benzo[a]pyrene showed a positive correlation with ∑PUFA (r = 0.758) and PUFA/SFA (r = 0.779). Unsaturated fatty acids have a significant impact on the formation of PAHs in heat-treated food, as confirmed by a study by Lee et al. [43]. The PAH that had the highest correlation with fatty acids was benzo[g,h,i]perylene BghiP.

There was no statistically significant correlation between the basic composition (content of: water, fat, protein, connective tissue and salt) and the fatty acid profile. Similar results were obtained when analyzing the correlation of TBARS value and fatty acids.

## 4. Conclusions

Grilling can lead to contamination of food by polycyclic aromatic hydrocarbons (PAHs) for which toxic, mutagenic and carcinogenic effects have been evidenced. For this reason, it is important to monitor the level of polycyclic hydrocarbons in food and to look for solutions to reduce their level in food products.

Partial replacement of animal tallow with sunflower oil, canola oil, linseed oil, olive oil and milk fat affected the physical properties, oxidative stability, fatty acid profile and PAH levels in beef burgers. Partial replacement of beef tallow with vegetable oils and milk fat contributed to a change in the L* and a* color components on the burger surface and a reduction in hardness and chewiness. However, no effect of fat substitution was observed on the burger elasticity and b* color component. The susceptibility of acids to oxidize has been increased in proportion to the number of unsaturated bonds in each fatty chain. Burgers with canola oil (CO) and linseed oil (LO) were the most susceptible to oxidation, whereas burgers with reduced fat content (CON_LOW FAT) were the most stable in terms of oxidation.

The highest ∑12PAH content was found in samples with canola oil (105.5 mg/kg), where the highest monounsaturated fatty acids (PUFAs) were also present, and in the control group without substitution, where high levels of saturated fat (40.34%) and *trans*-fat conformations were found. Studies confirm that partial replacement of beef tallow with oils and milk fat and reduction in fat content in burgers to be grilled can be an effective way to change their fatty acid profile and reduce the cyclization reaction of organic compounds causing PAH formation. Analysis of correlation coefficients showed that there is a relationship between the fatty acid profile and the presence of selected PAHs, especially between the content of fluorene, benz[a]anthracene, benzo[b]fluoranthene and benzo[k]fluoranthene and fatty acids such as γ-linolenic acid GLA (C18:3 6,9,12), eicosenoic acid (C20:1), erucic acid (C22:1) and nervonic acid (C24:1). The results of this study indicate that the substitution of beef tallow with selected vegetable oils and milk fat may be a promising approach in designing meat burgers with a more favorable fatty acid profile and lower PAH values compared with conventional products.

## Figures and Tables

**Table 1:** The recipe ingredients of the control group beef burgers (CON), burgers with reduced fat (CON_LOW FAT), burgers with fat substitutions, i.e., sunflower oil (SO), canola oil (CO), linseed (LO), olive oil (OO) and milk fat (MF).

Component (%)	CON	CON_LOW FAT	SO	CO	LO	OO	MF
beef neck	73.2	82.5	73.2	73.2	73.2	73.2	73.2
beef tallow	19.3	10.0	10.3	10.3	10.3	10.3	10.3
water	5.0	5.0	5.0	5.0	5.0	5.0	5.0
black pepper	0.4	0.4	0.4	0.4	0.4	0.4	0.4
salt	1.1	1.1	1.1	1.1	1.1	1.1	1.1
rice protein preparation	1.0	1.0	1.0	1.0	1.0	1.0	1.0
fat substitute	0	0	9.0	9.0	9.0	9.0	9.0

**Table 2:** Proximate composition of grilled burgers on the 1st and 6th day of storage (mean ± SE).

	Moisture		Fat		Protein		Salt		Con. Tiss	
	Day 1	Day 6	Day 1	Day 6	Day 1	Day 6	Day 1	Day 6	Day 1	Day 6
CON	50.12 ± 0.22 ^Eb^	55.05 ± 0.58 ^Da^	14.69 ± 0.15 ^B^	15.23 ± 0.48 ^B^	28.75 ± 0.16 ^BCa^	25.89 ± 0.34 ^Db^	1.42 ± 0.04 ^Ab^	0.39 ± 0.05 ^Eb^	4.33 ± 0.20 ^Aa^	3.72 ± 0.11 ^Ab^
CON_LOW FAT	50.99 ± 0.30 ^Db^	59.25 ± 0.04 ^Aa^	12.16 ± 0.09 ^Da^	10.28 ± 0.16 ^Fb^	30.37 ± 0.15 ^Aa^	25.78 ± 0.11 ^Db^	0.68 ± 0.09 ^Db^	1.20 ± 0.03 ^Ca^	3.63 ± 0.03 ^CDa^	2.35 ± 0.15 ^Cb^
SO	50.52 ± 0.16 ^BDb^	51.26 ± 0.15 ^Ea^	15.27 ± 0.26 ^A^	15.12 ± 0.01 ^BC^	28.19 ± 0.14 ^Db^	29.31 ± 0.21 ^Aa^	0.45 ± 0.02 ^Eb^	0.95 ± 0.06 ^Da^	3.99 ± 0.14 ^ABCa^	3.16 ± 0.04 ^Bb^
CO	49.09 ± 0.30 ^Fb^	56.71 ± 0.24 ^Ca^	14.30 ± 0.04 ^Ba^	12.88 ± 0.08 ^Db^	29.28 ± 0.22 ^Ba^	26.66 ± 0.06 ^Cb^	0.61 ± 0.02 ^Eb^	1.62 ± 0.08 ^Ba^	4.20 ± 0.11 ^ABa^	3.16 ± 0.06 ^Bb^
LO	53.83 ± 0.04 ^Ba^	51.83 ± 0.30 ^Eb^	13.36 ± 0.06 ^Cb^	17.75 ± 0.34 ^Aa^	26.21 ± 0.26 ^Fb^	27.34 ± 0.16 ^Ba^	1.16 ± 0.07 ^Bb^	1.95 ± 0.18 ^Aa^	4.16 ± 0.11 ^ABa^	3.80 ± 0.15 ^Ab^
OO	54.74 ± 0.22 ^A^	54.51 ± 0.09 ^D^	12.23 ± 0.16 ^Db^	14.48 ± 0.08 ^Ca^	27.29 ± 0.23 ^E^	27.39 ± 0.06 ^B^	1.25 ± 0.14 ^ABb^	1.61 ± 0.08 ^Ba^	3.90 ± 0.19 ^BCDa^	3.10 ± 0.09 ^Bb^
MF	52.13 ± 0.04 ^Cb^	58.32 ± 0.08 ^Ba^	12.01 ± 0.11 ^D^	11.93 ± 0.11 ^E^	28.43 ± 0.15 ^BCa^	26.66 ± 0.06 ^Cb^	0.95 ± 0.01 ^Cb^	1.59 ± 0.05 ^Ba^	3.60 ± 0.06 ^Da^	1.82 ± 0.09 ^Db^
SEM	0.426	0.633	0.279	0.512	0.280	0.249	0.088	0.110	0.063	0.148
Effects:										
Treatment	***		***		***		***		***	
Storage time	***		***		***		***		***	
Treatment × Storage time	***		***		***		***		***	

CON—control group beef burgers, CON_LOW FAT—burgers with reduced fat, burgers with fat substitutions, i.e., sunflower oil (SO), canola oil (CO), linseed (LO), olive oil (OO) and milk fat (MF). (^A–F^)—means with different letters showing significant effect of treatment group in the same day of storage; (^a,b^)—means with different letters showing significant effect of storage time in each treatment group; *p* ≤ 0.05. Effects *** *p* ≤ 0.001; NS—no significant; SE—standard error of the mean.

**Table 3:** CIE parameters (L*, a* and b*) in raw and grilled burgers (mean ± SE).

	Sample	L* [-]		a* [-]		b* [-]	
		Day 1	Day 6	Day 1	Day 6	Day 1	Day 6
*Raw burgers*	CON	46.65 ± 2.18 ^B^	46.60 ± 2.13 ^C^	19.40 ± 2.38	19.37 ± 2.34	10.46 ± 1.85 ^C^	10.42 ± 1.77 ^C^
	CON_LOW FAT	47.66 ± 2.33 ^B^	47.85 ± 2.34 ^CB^	22.17 ± 3.67	22.27 ± 3.84	13.15 ± 2.12 ^B^	12.92 ± 1.83 ^B^
	SO	52.73 ± 2.92 ^A^	53.69 ± 3.37 ^A^	19.01 ± 2.53	19.41 ± 2.53	15.78 ± 1.87 ^A^	16.43 ± 2.45 ^A^
	CO	51.30 ± 2.13 ^A^	51.61 ± 2.31 ^A^	20.52 ± 2.13	21.10 ± 1.97	14.69 ± 1.85 ^AB^	15.04 ± 1.82 ^AB^
	LO	51.44 ± 1.89 ^A^	50.75 ± 2.35 ^AB^	18.93 ± 2.48	19.45 ± 2.10	16.41 ± 1.64 ^A^	16.53 ± 1.57 ^A^
	OO	50.75 ± 2.00 ^A^	50.69 ± 1.98 ^AB^	19.52 ± 1.95	19.48 ± 1.91	16.14 ± 1.10 ^A^	16.10 ± 1.06 ^A^
	MF	52.35 ± 2.24 ^A^	52.29 ± 2.19 ^A^	20.29 ± 2.08	20.24 ± 1.99	15.66 ± 1.41 ^A^	15.62 ± 1.38 ^A^
SEM		0.3671	0.392	0.315	0.310	0.309	0.322
Effects							
Treatment		***		**		***	
Storage time		NS		NS		NS	
Treatment × Storage time		NS		NS		NS	
*Grilled burgers*	CON	35.32 ± 0.87 ^A^	35.16 ± 1.03 ^A^	7.30 ± 0.78 ^B^	7.33 ± 0.80 ^BC^	11.94 ± 0.62 ^A^	11.69 ± 0.56 ^A^
	CON_LOW FAT	31.04 ± 1.27 ^C^	30.72 ± 1.49 ^CD^	9.05 ± 1.02 ^A^	8.82 ± 1.09 ^AB^	9.73 ± 0.97 ^BC^	9.56 ± 0.82 ^BCD^
	SO	28.09 ± 2.77 ^C^	27.32 ± 2.73 ^E^	7.26 ± 1.95 ^B^	6.84 ± 1.92 ^C^	8.51 ± 2.04 ^C^	7.99 ± 2.08 ^D^
	CO	31.72 ± 2.37 ^BC^	31.50 ± 2.01 ^BC^	8.77 ± 0.91 ^AB^	8.94 ± 0.97 ^A^	10.02 ± 1.22 ^BC^	10.09 ± 1.28 ^ABC^
	LO	31.13 ± 1.60 ^A^	31.71 ± 2.16 ^BC^	9.07 ± 1.18 ^A^	8.82 ± 0.95 ^AB^	10.56 ± 1.07 ^AB^	10.33 ± 0.98 ^AB^
	OO	34.17 ± 1.67 ^AB^	33.89 ± 1.89 ^AB^	8.50 ± 0.88 ^AB^	8.84 ± 0.86 ^AB^	10.03 ± 0.90 ^BC^	10.26 ± 0.89 ^AB^
	MF	27.64 ± 2.08 ^C^	27.93 ± 2.54 ^DE^	7.30 ± 1.20 ^B^	6.92 ± 1.16 ^C^	8.72 ± 1.29 ^C^	8.55 ± 1.34 ^CD^
SEM		0.384	0.396	0.166	0.172	0.190	0.196
Effects:							
Treatment		***		***		***	
Storage time		NS		NS		NS	
Treatment × Storage time		NS		NS		NS	

CON—control group beef burgers, CON_LOW FAT—burgers with reduced fat, burgers with fat substitutions, i.e., sunflower oil (SO), canola oil (CO), linseed (LO), olive oil (OO) and milk fat (MF). (^A–E^)—means with different letters showing significant effect of treatment group in the same day of storage; Effects ** *p* ≤ 0.01, *** *p* ≤ 0.001; NS—no significant; SE—standard error of the mean.

**Table 4:** Textural properties of grilled burgers at 1st and 6th day of storage (mean ± SE).

	SPRINGINESS [-]		CHEWINESS [N]		HARDNESS [N]	
	Day 1	Day 6	Day 1	Day 6	Day 1	Day 6
CON	0.68 ± 0.06	0.62 ± 0.04	30.79 ± 3.39 ^Ba^	34.93 ± 2.93 ^Bb^	59.3 ± 4.51 ^Ba^	74.88 ± 7.99 ^BCb^
CON_LOW FAT	0.66 ± 0.04 ^b^	0.59 ± 0.06 ^a^	45.33 ± 5.10 ^Db^	34.12 ± 4.32 ^Ba^	88.64 ± 9.82 ^D^	76.72 ± 12.79 ^C^
SO	0.67 ± 0.05	0.67 ± 0.05	38.97 ± 4.99 ^Cb^	28.58 ± 6.79 ^ABa^	80.48 ± 9.49 ^CDb^	57.77 ± 14.99 ^ABCa^
CO	0.67 ± 0.05	0.65 ± 0.05	34.43 ± 3.11 ^BCb^	22.75 ± 3.29 ^Aa^	73.65 ± 7.61 ^Cb^	45.71 ± 5.95 ^Aa^
LO	0.68 ± 0.07	0.58 ± 0.11	18.94 ± 1.94 ^Aa^	31.88 ± 4.73 ^Bb^	34.99 ± 3.23 ^Aa^	66.46 ± 12.90 ^BCb^
OO	0.66 ± 0.03	0.60 ± 0.09	18.85 ± 1.72 ^Aa^	27.10 ± 5.16 ^ABb^	39.87 ± 4.61 ^Aa^	55.69 ± 10.38 ^ABb^
MF	0.67 ± 0.07	0.62 ± 0.10	22.99 ± 1.47 ^Aa^	30.46 ± 3.99 ^ABb^	45.62 ± 1.78 ^Aa^	66.80 ± 11.57 ^BCb^
SEM	0.0079	0.0119	1.5616	0.8941	3.1910	2.2624
Effects:						
Treatment	NS		***		***	
Storage time	***		NS		NS	
Treatment × Storage time	NS		***		***	

CON—control group beef burgers, CON_LOW FAT—burgers with reduced fat, burgers with fat substitutions, i.e., sunflower oil (SO), canola oil (CO), linseed (LO), olive oil (OO) and milk fat (MF). (^A–D^)—means with different letters showing significant effect of treatment group in the same day of storage; (^a,b^)—means with different letters showing significant effect of storage time in each treatment group; *p* ≤ 0.05. Effects *** *p* ≤ 0.001; NS—no significant; SE—standard error of the mean.

**Table 5:** Fatty acid profile of raw burgers on day 1 and day 6 of storage (mean ± SE).

Fatty Acids	CON	CON_LOW FAT	SO	CO	LO	OO	MF	CON	CON_LOW FAT	SO	CO	LO	OO	MF	SEM		Effects	
	Day 1							Day 6								Treatment	Storage Time	Treatment × Storage Time
C12:0	0.06 ^B^	0.06 ^B^	0.04 ^ABb^	0.03 ^A^	0.03 ^A^	0.03 ^Ab^	1.28 ^C^	0.06 ^A^	0.05 ^A^	0.03 ^Aa^	0.03 ^A^	0.03 ^A^	0.02 ^Aa^	1.35 ^B^	0.070	***	NS	NS
C14:0	2.66 ^Db^	2.8 ^Eb^	1.96 ^Cb^	1.52 ^B^	1.29 ^A^	1.32 ^Ab^	6.20 ^F^	2.47 ^Ba^	2.43 ^Ba^	1.36 ^Aa^	1.57 ^A^	1.36 ^A^	1.19 ^Aa^	6.42 ^C^	0.259	***	**	***
C14:1	1.76 ^CD^	1.68 ^CD^	1.45 ^Cb^	0.95 ^B^	0.62 ^A^	0.79 ^ABb^	1.93 ^E^	1.48 ^B^	1.44 ^B^	0.82 ^Aa^	0.94 ^A^	0.68 ^A^	0.64 ^Aa^	1.86 ^C^	0.075	***	***	***
C15:0	0.33 ^C^	0.34 ^C^	0.24 ^B^	0.19 ^AB^	0.18 ^A^	0.18 ^A^	0.68 ^D^	0.32 ^B^	0.35 ^B^	0.20 ^A^	0.21 ^A^	0.21 ^A^	0.21 ^A^	0.71 ^C^	0.027	***	NS	NS
C15:1	0.23 ^BC^	0.2 ^B^	0.15 ^A^	0.13 ^A^	0.13 ^A^	0.12 ^A^	0.26 ^C^	0.20 ^B^	0.22 ^B^	0.12 ^A^	0.13 ^A^	0.14 ^A^	0.11 ^A^	0.26 ^C^	0.008	***	NS	NS
C16:0	24.36 ^C^	25.06 ^C^	18.42 ^B^	16.13 ^A^	16.19 ^A^	18.71 ^Bb^	28.48 ^D^	24.73 ^B^	24.46 ^B^	16.74 ^A^	16.76 ^A^	16.48 ^A^	15.87 ^Aa^	28.85 ^C^	0.743	***	*	**
C16:1	6.16 ^D^	5.67 ^D^	4.93 ^Cb^	3.39 ^B^	2.50 ^A^	3.22 ^ABb^	4.78 ^C^	5.76 ^D^	5.01 ^CD^	3.24 ^ABa^	3.52 ^AB^	2.54 ^A^	2.79 ^Aa^	4.33 ^BC^	0.194	***	***	**
C17:0	0.73 ^B^	0.85 ^B^	0.50 ^A^	0.48 ^A^	0.48 ^A^	0.43 ^A^	0.74 ^B^	0.80 ^B^	0.92 ^C^	0.46 ^A^	0.49 ^A^	0.51 ^A^	0.41 ^A^	0.75 ^B^	0.027	***	NS	NS
C17:1	0.98 ^D^	0.99 ^D^	0.72 ^C^	0.59 ^B^	0.51 ^A^	0.53 ^AB^	0.75 ^C^	0.98 ^C^	0.95 ^C^	0.57 ^A^	0.60 ^A^	0.50 ^A^	0.49 ^A^	0.72 ^B^	0.030	***	**	*
C18:0	11.51 ^D^	11.47 ^D^	8.20 ^AB^	7.37 ^A^	9.37 ^BCa^	8.10 ^AB^	10.6 ^CD^	12.35 ^CD^	14.16 ^D^	8.52 ^AB^	7.63 ^A^	9.98 ^ABCb^	8.00 ^A^	10.91 ^BC^	0.324	***	**	NS
C18:1 trans	0.01 ^CD^	0.01 ^CD^	0.01 ^BC^	0 ^A^	0.02 ^E^	0.01 ^B^	0.02 ^DE^	0.01 ^CD^	0.02 ^DE^	0.01 ^BC^	0 ^A^	0.02 ^E^	0.01 ^B^	0.02 ^DE^	0.001	***	NS	NS
C18:1 cis	45.42 ^D^	45.39 ^D^	41.34 ^Cb^	53.79 ^Eb^	29.61 ^A^	58.83 ^Fb^	36.7 ^B^	44.88 ^C^	43.82 ^C^	37.19 ^Ba^	53.04 ^Da^	29.87 ^A^	43.15 ^Ca^	36.00 ^B^	1.298	***	***	***
C18:2 9,12 trans	1.17 ^B^	1.20 ^B^	0.99 ^B^	0.60 ^A^	0.49 ^A^	0.52 ^A^	1.03 ^B^	1.12 ^B^	1.10 ^B^	0.66 ^A^	0.62 ^A^	0.47 ^A^	0.53 ^A^	1.03 ^B^	0.045	***	**	**
C18:2 9,12 cis	2.79 ^A^	1.88 ^Aa^	18.63 ^Da^	9.73 ^C^	34.37 ^E^	4.92 ^Bb^	1.99 ^A^	2.20 ^A^	2.25 ^Ab^	27.68 ^Cb^	9.41 ^B^	33.22 ^D^	3.38 ^Aa^	1.99 ^A^	1.847	***	*	***
C18:3 6,9,12	0.28 ^C^	0.29 ^C^	0.19 ^B^	0.36 ^D^	0.15 ^A^	0.13 ^Aa^	0.26 ^C^	0.26 ^B^	0.25 ^B^	0.14 ^A^	0.34 ^C^	0.14 ^A^	0.5 ^Db^	0.24 ^B^	0.016	***	***	***
C18:3 9,12,15	0.36 ^B^	0.30 ^AB^	0.24 ^A^	2.61 ^Db^	3.13 ^E^	0.51 ^C^	0.35 ^B^	0.35 ^AB^	0.32 ^AB^	0.23 ^A^	2.45 ^Ca^	2.98 ^D^	0.57 ^B^	0.35 ^AB^	0.174	***	NS	NS
C20:0	0.59 ^C^	0.59 ^C^	0.51 ^B^	0.56 ^C^	0.32 ^A^	0.48 ^B^	0.67 ^D^	0.53 ^CD^	0.56 ^CD^	0.43 ^AB^	0.55 ^CD^	0.34 ^A^	0.47 ^AB^	0.67 ^D^	0.017	***	NS	NS
C20:1	0.55 ^Cb^	0.55 ^C^	0.51 ^Cb^	0.84 ^Db^	0.26 ^A^	0.38 ^ABb^	0.48 ^BC^	0.44 ^Ba^	0.42 ^B^	0.31 ^ABa^	0.74 ^Ca^	0.25 ^A^	0.31 ^ABa^	0.42 ^B^	0.027	***	***	NS
C20:2 + C21:0	0.10 ^C^	0.10 ^C^	0.06 ^ABb^	0.08 ^ABC^	0.08 ^BCb^	0.05 ^A^	0.09 ^C^	0.09 ^CD^	0.11 ^D^	0.05 ^Aa^	0.07 ^ABC^	0.07 ^ABCa^	0.06 ^AB^	0.09 ^BCD^	0.003	***	NS	NS
C20:3 8,11,14	0.08 ^BC^	0.10 ^C^	0.06 ^AB^	0.09 ^C^	0.05 ^Ab^	0.05 ^A^	0.09 ^C^	0.10 ^C^	0.10 ^C^	0.05 ^AB^	0.08 ^ABC^	0.04 ^Aa^	0.08 ^BC^	0.09 ^C^	0.004	***	NS	*
C20:4 + C20:3 11,14,17	0.19 ^B^	0.15 ^AB^	0.11 ^A^	0.11 ^AB^	0.15 ^ABb^	0.12 ^AB^	0.15 ^AB^	0.27	0.26	0.12	0.12	0.11 ^a^	0.14	0.14	0.010	***	NS	NS
C22:0	0.02 ^A^	0.02 ^A^	0.22 ^D^	0.14 ^C^	0.07 ^B^	0.07 ^B^	0.04 ^A^	0.02 ^A^	0.02 ^A^	0.31 ^C^	0.13 ^B^	0.07 ^AB^	0.08 ^AB^	0.04 ^A^	0.013	***	*	**
C22:1	0.04 ^BC^	0.04 ^C^	0.02 ^AB^	0.14 ^D^	0.02 ^A^	0.02 ^A^	0.05 ^C^	0.03 ^B^	0.04 ^B^	0.02 ^A^	0.13 ^C^	0.02 ^A^	0.02 ^A^	0.05 ^B^	0.006	***	NS	NS
C20:5	0.03	0.03	0.02	0.02	0.02 ^b^	0.02	0.03	0.04 ^AB^	0.04 ^B^	0.02 ^AB^	0.02 ^A^	0.02 ^Aa^	0.02 ^A^	0.04 ^AB^	0.002	***	NS	NS
C24:0	0.08 ^A^	0.08 ^A^	0.13 ^B^	0.11 ^B^	0.1 ^AB^	0.32 ^C^	0.08 ^A^	0.09 ^A^	0.07 ^A^	0.15 ^A^	0.1 ^A^	0.09 ^A^	0.28 ^B^	0.07 ^A^	0.012	***	NS	NS
C24:1	0 ^A^	0 ^A^	0 ^A^	0.04 ^B^	0 ^A^	0 ^A^	0 ^A^	0 ^A^	0 ^A^	0 ^A^	0.04 ^B^	0 ^A^	0 ^A^	0 ^A^	0.002	***	NS	**
C22:6	0.10 ^C^	0.09 ^BC^	0.06 ^AB^	0.06 ^AB^	0.07 ^ABCb^	0.05 ^A^	0.08 ^ABC^	0.13 ^C^	0.12 ^BC^	0.06 ^A^	0.05 ^A^	0.05 ^Aa^	0.05 ^A^	0.08 ^AB^	0.005	***	NS	NS

CON—control group beef burgers, CON_LOW FAT— burgers with reduced fat, burgers with fat substitutions, i.e., sunflower oil (SO), canola oil (CO), linseed (LO), olive oil (OO) and milk fat (MF); SFA = saturated fatty acids; MUFA = monounsaturated fatty acids; PUFA = polyunsaturated fatty acids. (^A–F^)—means with different letters showing significant effect of treatment group in the same day of storage; (^a,b^)—means with different letters showing significant effect of storage time in each treatment group; *p* ≤ 0.05. Effects * *p* ≤ 0.05, ** *p* ≤ 0.01, *** *p* ≤ 0.001; NS—no significant; SE—standard error of the mean.

**Table 6:** The sum of SFA, MUFA, and PUFA burgers on day 1 and day 6 of storage (mean ± SE).

Fatty Acids	CON	CON_LOW FAT	SO	CO	LO	OO	MF	CON	CON_LOW FAT	SO	CO	LO	OO	MF	SEM		Effects	
	Day 1							Day 6								Treatment	Storage Time	Treatment × Storage Time
∑SFA	40.34 ^C^	41.28 ^C^	30.22 ^B^	26.53 ^Aa^	28.02 ^AB^	29.64 ^Bb^	48.76 ^D^	41.37 ^B^	43.02 ^B^	28.20 ^A^	27.48 ^Ab^	29.06 ^A^	26.53 ^Aa^	49.77 ^C^	1.303	***	NS	**
∑MUFA	55.15 ^D^	54.54 ^D^	49.14 ^Cb^	59.88 ^Eb^	33.66 ^A^	63.89 ^Fb^	44.96 ^B^	53.78 ^D^	51.91 ^D^	42.29 ^Ba^	59.14 ^Ea^	34.02 ^A^	47.52 ^Ca^	43.65 ^BC^	1.388	***	***	***
∑PUFA	5.09 ^AB^	4.15 ^A^	20.37 ^Da^	13.67 ^C^	38.52 ^E^	6.36 ^Bb^	4.06 ^A^	4.55 ^A^	4.55 ^A^	29.00 ^Cb^	13.15 ^B^	37.09 ^D^	5.32 ^Aa^	4.04 ^A^	1.912	***	NS	***
PUFA/SFA	0.13 ^B^	0.10 ^ABa^	0.67 ^Ea^	0.52 ^Db^	1.37 ^F^	0.21 ^C^	0.08 ^A^	0.11 ^A^	0.11 ^Ab^	1.03 ^Cb^	0.48 ^Ba^	1.28 ^C^	0.20 ^A^	0.08 ^A^	0.070	***	NS	***
n6/n3	6.65 ^ABb^	5.44 ^AB^	57.80 ^Ca^	3.79 ^A^	10.75 ^Ba^	8.93 ^ABb^	5.18 ^ABb^	5.27 ^Aa^	5.70 ^A^	92.76 ^Bb^	3.92 ^A^	10.99 ^Ab^	6.28 ^Aa^	5.03 ^Aa^	3.942	***	***	***

CON—control group beef burgers, CON_LOW FAT—burgers with reduced fat, burgers with fat substitutions, i.e., sunflower oil (SO), canola oil (CO), linseed (LO), olive oil (OO) and milk fat (MF); SFA = saturated fatty acids; MUFA = monounsaturated fatty acids; PUFA = polyunsaturated fatty acids. (^A–F^)—means with different letters showing significant effect of treatment group in the same day of storage; (^a,b^)—means with different letters showing significant effect of storage time in each treatment group; *p* ≤ 0.05; Effects ** *p* ≤ 0.01, *** *p* ≤ 0.001; NS—no significant; SE—standard error of the mean.

**Table 7:** PAHs formation in grilled burgers on day 1 and day 6 of storage (mean ± SE).

PAH Compound	PAH Concentration (mg/kg) Day 1							PAH Concentration (mg/kg) Day 6									Effects:		
	CON	CON_LOWFAT	SO	CO	LO	OO	MF	SEM	CON	CON_LOW FAT	SO	CO	LO	OO	MF	SEM	Treatment	Storage Time	Treatment × Storage Time
Fluorene	0.39 ^B^	0.29 ^B^	0.28 ^B^	0.94 ^C^	0.41 ^B^	0 ^A^	0.38 ^B^	0.060	0.35 ^B^	0.23 ^B^	0.35 ^B^	0.73 ^C^	0.33 ^B^	0 ^A^	0.29 ^B^	0.047	***	*	NS
Anthracene	0.51 ^A^	0.38 ^A^	1.14 ^CD^	1.49 ^D^	0.96 ^BCa^	0.54 ^Ab^	0.68 ^AB^	0.087	0.43 ^B^	0.37 ^B^	1.02 ^CD^	1.56 ^E^	1.17 ^Db^	0 ^Aa^	0.81 ^C^	0.112	***	NS	***
Fluoranthene	2.09 ^BCD^	2.03 ^BCD^	1.57 ^B^	0.88 ^Ab^	1.76 ^BCa^	2.28 ^CD^	2.47 ^Db^	0.116	2.04 ^B^	1.87 ^B^	1.84 ^B^	0 ^Ab^	2.02 ^Bb^	1.87 ^B^	2.12 ^Ba^	0.157	***	**	***
Benzo[b]fluorine	23.21 ^D^	14.34 ^A^	21.44 ^CD^	22.11 ^D^	18.74 ^B^	19.9 ^BC^	17.92 ^B^	0.637	22.99 ^D^	13.99 ^A^	22.67 ^D^	21.37 ^C^	17.86 ^B^	18.51 ^B^	18.23 ^B^	0.666	***	NS	*
Benz[a]anthracene	2.09 ^C^	1.52 ^B^	2.22 ^C^	0.47 ^A^	2.13 ^C^	2.36 ^C^	1.37 ^Bb^	0.142	1.79 ^CD^	1.63 ^C^	2.07 ^DE^	0.35 ^A^	2.28 ^E^	2.2 ^E^	1.02 ^Ba^	0.148	***	*	NS
Chrysene	16.48 ^C^	9.37 ^A^	12.1 ^B^	18.86 ^D^	13.44 ^B^	15.27 ^Cb^	15.7 ^C^	0.652	16.21 ^D^	8.87 ^A^	13.17 ^B^	19.12 ^E^	12.87 ^B^	14.12 ^Ca^	14.98 ^C^	0.658	***	NS	**
Σlight PAHs	44.77 ^D^	27.93 ^Ab^	38.75 ^BCa^	44.75 ^D^	37.44 ^B^	40.35 ^Cb^	38.52 ^BC^	1.184	43.81 ^D^	26.96 ^Aa^	41.12 ^Cb^	43.13 ^D^	36.53 ^B^	36.7 ^Ba^	37.45 ^B^	1.190	***	***	***
Benzo[b]fluoranthene	1.35 ^Eb^	0.63 ^C^	0.25 ^ABb^	2.47 ^F^	0 ^A^	0.41 ^BCb^	0.96 ^D^	0.175	1.09 ^Ca^	0.49 ^B^	0 ^Aa^	2.12 ^D^	0 ^A^	0 ^Aa^	1.11 ^C^	0.167	***	***	***
Benzo[k]fluoranthene	2.47 ^C^	1.37 ^B^	2.14 ^C^	4.86 ^Db^	0 ^A^	0 ^A^	0 ^A^	0.373	2.23 ^C^	1.29 ^B^	2.31 ^C^	4.15 ^Da^	0 ^A^	0 ^A^	0 ^A^	0.328	***	**	***
Benzo[a]pyrene	2.7 ^C^	1.23 ^A^	1.96 ^B^	3.3 ^D^	4.21 ^Eb^	1.93^B^	1.97 ^B^	0.211	2.44 ^C^	1.52 ^A^	1.77 ^AB^	3.27 ^D^	3.19 ^Da^	2.16 ^BC^	2.13 ^BC^	0.141	***	*	***
Dibenz[a,h]anthracene	39.14 ^D^	16.87 ^A^	21.81 ^B^	46.97 ^E^	36.63 ^C^	20.18 ^Bb^	14.64 ^A^	2.624	37.06 ^D^	17.13 ^B^	21.63 ^C^	45.76 ^E^	37.22 ^D^	15.23 ^Aa^	15.32 ^A^	2.619	***	**	***
Benzo[g,h,i]perylene	0.79 ^B^	0.43 ^AB^	0.58 ^Ba^	3.15 ^Ea^	2.43 ^Da^	1.43 ^C^	0 ^A^	0.240	0.89B ^C^	0.29 ^AB^	0.79 ^Bb^	4.26 ^Eb^	2.98 ^Db^	1.13 ^C^	0 ^A^	0.323	***	***	***
Indeno [1,2,3-cd]pyrene	ND	ND	ND	ND	ND	ND	ND	0.000	ND	ND	ND	ND	ND	ND	ND	0.000	ND	ND	ND
Σheavy PAHs	46.45 ^Cb^	20.53 ^A^	26.74 ^B^	60.75 ^D^	43.27 ^C^	23.95 ^Bb^	17.57 ^A^	3.346	43.71 ^Da^	20.72 ^B^	26.5 ^C^	59.56 ^E^	43.39 ^D^	18.52 ^Aa^	18.56 ^A^	3.318	***	***	***
Σ12 PAHs	91.22 ^Eb^	48.46 ^A^	65.49 ^C^	105.5 ^Fb^	80.71 ^D^	64.3 ^Cb^	56.09 ^B^	4.205	87.52 ^Ea^	47.68 ^A^	67.62 ^C^	102.69 ^Fa^	79.92 ^D^	55.22 ^Ba^	56.01 ^B^	4.132	***	***	***

CON—control group beef burgers, CON_LOW FAT—burgers with reduced fat, burgers with fat substitutions, i.e., sunflower oil (SO), canola oil (CO), linseed (LO), olive oil (OO) and milk fat (MF); (^A–F^)—means with different letters showing significant effect of treatment group in the same day of storage; (^a,b^)—means with different letters showing significant effect of storage time in each treatment group; *p* ≤ 0.05. Effects * *p* ≤ 0.05, ** *p* ≤ 0.01, *** *p* ≤ 0.001; ND—not detected; NS—no significant; SE—standard error of the mean.

**Table 8:** Correlation coefficients between the fatty acid profile of raw burgers and PAHs of grilled burgers.

PAHs	Fatty Acids												
	C14:1	C16:0	C18:0	C18:2 9,12 trans	C18:3 6,9,12	C18:3 9,12,15	C20:1	C22:0	C22:1	C24:1	∑SFA	∑PUFA	PUFA/SFA
F					0.758				0.894	0.869			
ANT			−0.764					0.772					
FL										−0.823			
BbF													
BaA					−0.840		−0.807		−0.941	−0.835			
ChR													
BbFL					0.858		0.903		0.920	0.844			
BkF					0.765		0.907		0.806	0.812			
BaP						0.897						0.758	0.779
BghiP	−0.828	−0.829		−0.800		0.897					−0.804		
Moisture	0.045	0.274	0.366	0.056	0.057	−0.113	−0.225	−0.359	−0.027	−0.075	0.313	−0.294	−0.298
Fat	−0.395	−0.341	−0.151	−0.383	0.156	0.180	−0.266	0.28	0.015	0.131	−0.296	0.248	0.260
Protein	0.172	−0.021	−0.264	0.143	0.091	−0.188	0.335	0.331	0.124	0.087	−0.072	−0.038	−0.031
Salt	−0.307	−0.154	−0.069	−0.421	0.057	0.323	−0.329	−0.214	−0.039	−0.014	−0.106	0.160	0.164
Con. Tiss	−0.228	−0.382	−0.32	−0.195	−0.148	0.329	0.173	0.08	−0.001	0.109	−0.430	0.316	0.324
TBARS	−0.433	−0.341	−0.151	−0.383	0.156	0.180	−0.266	0.28	0.015	0.131	−0.296	0.248	0.260

F—fluorene; ANT—anthracene; FL—fluoranthene; BbF—benzo[b]fluorine; BaA—benz[a]anthracene; CHR—chrysene; BbFL—benzo[b]fluoranthene; BkF—benzo[k]fluoranthene; BaP—benza[a]pyrene; BghiP—benzo[g,h,i]perylene.

## Data Availability

The data presented in this study are available on request from the corresponding author.
